# Medical Opinion and Movement

**Published:** 1911-04-29

**Authors:** 


### Medical Opinion and Movement.

### Fatty Heart.

In the *Wiener Klinische Wochenschrift*, Dr. Schwarz draws attention to a condition of fatty heart which may be made evident by radioscopic examination. In the radiograph of a normal individual, between the shadow of the apex and of the left ventricle, describing a parabolic line on the one hand and the diaphragm on the other, there is a triangular space, the extent of which varies with the movements of the heart and of the diaphragm, and which is filled by the lung. This latter allows the cathodic rays to pass through and appears clear. By employing specially soft tubes, Dr. Schwarz has observed in obese individuals, and even in certain individuals not particularly fat, this space filled by a shadow less deep than that of the heart and not diminished by the cardiac movements. According to the author, this shadow is due to pericardial fat, and he has been able to confirm this idea by autopsies on a certain number of these cases. The author points out that it is important to recognise the fact, as otherwise this shadow might easily be mistaken for a considerable hypertrophy of the left ventricle.

### Thoracic Aneurysm.

Dr. Dmitrenko draws attention to an important sign of thoracic aneurysm already enunciated by Dr. Williamson—namely, a difference in the arterial blood pressure on the two sides of the body. According to Dr. Williamson, whenever the difference between the arterial blood-pressure of the two brachial arteries attains or exceeds 30 millimetres, there is strong presumption in favour of the existence of an aneurysm. Depending chiefly on this sign, Dr. Dmitrenko was able to diagnose aneurysm of the thoracic aorta in a woman aged forty-nine, admitted into hospital for pains which she had experienced for two years in the back, between the shoulder-blades and in the chest. The blood pressure measured 125 millimetres of mercury in the left brachial artery, and 155 millimetres in the right. Radioscopic examination fully confirmed the diagnosis. In another case of a woman aged fifty-four, complaining of pains in the back, dyspnœa, dysphagia, and frequent vomiting immediately after food, estimation of the blood pressure showed 200 millimetres for the right brachial artery and 235 millimetres for the left. Radioscopic examination showed the presence of an enormous dilatation of the whole of the ascending part of the aorta, with aneurysm of the transverse and descending portions.

### Temporary Arrest of the Heart.

At a meeting of the Belgian Academy of Medicine an interesting report was communicated by Dr. Thiriar on the post-mortem results of a fatal case of chloroform syncope. The patient was a young man whose heart suddenly failed during chloroform anæsthesia. By means of massage of the heart, the heart-beats reappeared after complete cessation for an hour. The patient showed various nervous symptoms (torpor, incontinence of urine, subnormal and then a raised temperature), and died eight days later. Histological examination, carried out for the first time in a case of this nature, showed that not a single nerve-cell had remained intact. There was venous thrombosis of the right kidney, neurosis of the convoluted tubules, and a diffuse hæmorrhagic pigmentation of the spleen. Dr. Sand, who made the examination, draws the following general conclusions: The human brain can survive a complete interruption of the circulation so long as it does not exceed twenty-five minutes. Beyond this it may resume its functions temporarily, but death is inevitable. In such cases of syncope, therefore, massage of the heart must be resorted to as quickly as possible. There is no case on record in which it has been efficacious after an interval of fifteen minutes, but when started within that time it has resulted in a complete and lasting cure thirteen times.

### Intoxications of Pregnancy.

An interesting case is recorded by Drs. Mayer and Linser in the *Münchener Medizinische Wochenschrift*, in which a severe condition of herpes gestationis was apparently completely cured by injections of serum from a healthy pregnant woman. The patient was a young woman, who in the course of her first pregnancy had an attack of herpes at the seventh month, accompanied by fever and severe general symptoms, and at the end of a fortnight was delivered of a weakly but healthy infant. Two weeks after confinement the skin affection disappeared. During the second pregnancy herpes appeared somewhat earlier, at the end of the fifth month. Beginning at the extremities it spread rapidly over the body, with formation of pustules and ulcers. The pain prevented sleep in spite of narcotics. The urine contained one to two parts per 1,000 albumen and casts. External and internal medication failed to relieve and abortion was contemplated, but there was the fear of puerperal fever from the septic condition of the eruption. Serum injections were, therefore, first tried. Ten cubic centimetres of serum from a normal woman were first injected intravenously in order to determine the effect of human serum upon the organism. As the result was negative, three days later 10 c.c. of serum from a healthy woman eight months pregnant were injected, and three days later another 20 c.c. were similarly injected. After the second injection the temperature fell steadily to normal in the course of three days, the vesicles dried up and no fresh ones appeared, so that at the end of a fortnight there was no trace of the skin affection. At the same time the albuminuria and casts disappeared from the urine. After five weeks' interval, however, a fresh eruption occurred. This readily yielded again to a further injection of 20 c.c. of serum, and the patient was delivered of a healthy child at full term. The authors suggest from the experience of this case that other conditions dependent upon pregnancy, such as persistent vomiting, eclampsia, albuminuria, etc., might similarly yield to this form of serum therapy.

### A New Local Anæsthetic.

Max Strauss, in the *Münchener Med. Woch.*, describes a new local anæsthetic which has been named cycloform and is the isobutylic ether of *p*-amidobenzoic acid. It consists of a white powder crystalline in form, slightly soluble in water, but readily so in alcohol, ether, and benzol. Average sized cats have taken as much as a gram of the substance by the mouth without obvious ill-effects. A saturated aqueous solution renders a rabbit's cornea insensitive in two minutes. A solution of 0.01 per cent. gives an anæsthesia comparable with that of cocaine. The drug has disinfectant properties also, as has been proved by experiment. The author has made use of it to dust over small recent wounds which need to be kept open, and also in the form of an ointment in the treatment of painful burns. He has found the ointment useful for chaps, eczema, intertrigo, inflamed hæmorrhoids, and painful ulcers of the leg. It can be used for a long time without local irritation and without losing its power, and it does not delay the healing of wounds.

### The Causation of Ozæna.

A long and interesting paper on ozæna is contributed to the *Journal of Laryngology, Rhinology and Otology* by J. S. Fraser and F. Esmond Reynolds, who, after discussing the question at length, come to the following conclusions: (*a*) No clear line of demarcation can be drawn between chronic purulent rhinitis and ozæna; (*b*) Both affections begin in early life as a hypertrophic catarrh of the nasal mucous membrane; the inferior turbinal is the most severely affected and has frequently gone on to atrophy, while the middle turbinal is still in the hypertrophic stage; (*c*) The most common causes are the exanthemata, coryza in infants, and syphilis; (*d*) Chronic purulent rhinitis leads to various changes in the nasal mucosa. In many cases there is atrophy of the turbinal bones, especially of the inferior turbinal. In some cases there is arterial disease, and in the majority there is sclerosis of the deeper layers of the submucous tissue; (*e*) Various organisms give rise to the first stages of ozæna, *i.e.* to acute and subacute purulent rhinitis, but the characteristic picture of ozæna is probably only produced when the bacillus mucosus ozænæ is present; (*f*) Ozæna is more likely to develop in a congenitally roomy nose than in a narrow one on account of the greater tendency in the former to stagnation and consequent putrefaction of the secretions; (*g*) Atrophy of nasal tissues may be due to pressure of the crusts and to vascular and sclerotic changes, but is probably mainly due to toxic influences; (*h*) Tubercle and syphilis are concerned in the production of ozæna in that they may lead to chronic purulent rhinitis; (*i*) Accessory sinus suppuration is not the cause of though it may complicate ozæna; (*k*) Ozæna often occurs in several members of the same family, and there are grounds for considering it a contagious disease; (*l*) A lowered state of general health and neglect of treatment have probably more to do with the transition of purulent rhinitis into ozæna than "congenital tissue weakness."

### The Parasitic Origin of Cancer.

M. Borrel recently read before the Paris Academy of Medicine a paper on the ætiology of cancer. He is of opinion that the only way of clearing up this vexed question is by the study of cancers in the very earliest stages of their development. He has been able to show the presence of worms in the cancerous growths removed from mice and rats, and considers these the cause of a still unknown cancer virus. In man, as regards cancers of the face, he incriminates the demodex as skin parasites capable of localising the disease. In former communications he showed that splinters and rusty needles may give rise to a cancer, and he called to mind cancers resulting from burns, chronic tuberculous and syphilitic lesions, and thinks that there is always a question of a superimposed infection. In the same way he believes that nævi and pigmented lesions are often the starting-point of malignant growths because cells of the pigmentary type can be receptive cells for the cancer virus. According to him statistics which show the frequency of cancer in certain districts are eloquent witnesses in favour of the parasitic theory of its origin. Cancer is rare in the Midi of France.

### Mammary Gigantism.

Dr. Henri Caubet contributes to the *Archives de médecine des enfants* an interesting article on that form of mammary hypertrophy met with about puberty. This variety of the disease is in many ways similar to that occurring during pregnancy but differs from it in some respects. It starts between the ages of eleven and sixteen, and appears to be an exaggeration of the normal mammary development. Hypertrophy is always rapid and in some cases a matter of days almost. Usually full growth takes months or even years. The size to which the breasts may attain is never as great as that met with in the other variety, but the organs may weigh as much as sixteen pounds, and measure 8 to 16 inches in length (reaching to the umbilicus or even the pubes), and 20 to 32 inches in circumference. Their distended skin is covered with dilated veins; the nipple is a large flattened pigmented patch. The organ is of uniform consistence in no way differing from the normal, and is painless on palpation. Apart from discomfort and respiratory embarrassment the condition gives rise to a rapid deterioration in the patients' general health, as evidenced by anorexia, progressive emaciation, pallor, and amenorrhœa. Unlike that of the pregnant woman it has no tendency to spontaneous regression, and if left untreated the patient dies from progressive enfeeblement. Although a pure hypertrophy, radical treatment is absolutely necessary in rapidly advancing cases, and amputation quickly restores the girl to health. In cases which develop slowly a mixed course of opotherapy may be tried with advantage, as owing to the connection which exists between the thyroid gland and the breasts there is some risk of myxœdema following amputation of the organs. (Such a result has been reported in the case of a male whose breasts were amputated for a hypertrophic enlargement). And again, no surgeon likes to submit a girl to a *diminutio formositatis* if this can be avoided. The author terms the condition "gigantisme du sein," which seems accurately to express the facts noticed. Its ætiology is unknown, but he reports the interesting fact that the mother of a case which came under his notice had in her youth submitted to amputation of both her breasts for the same affection, so that the disease may possibly be a familial or developmental one and not a purely accidental anomaly.

### Prophylactic Injections Prior to Operation.

At a recent meeting of the Paris Academy of Medicine, Dr. Doyen read a paper on the treatment of peritonitis and infective conditions by injections of *mycolysine*. As the result of recent experiments he has succeeded in preparing a solution which can safely be injected not only subcutaneously, but also intravenously and into the peritoneum. He states that it is sufficient to inject a few cubic centimetres of this fluid in the peritoneum twenty-four hours before a laparotomy, to prevent almost for certain all danger of a subsequent infection. In the grave infective diseases subcutaneous injection should be performed, and this may be supplemented in cases of immediate danger by an intravenous injection. The new colloidal solution may be diluted with isotonic salt solution, so as to form an artificial serum possessing the double properties of immunisation against almost every kind of infectious malady, and of feeding up the organism when given hypodermically. As much as four pints of this artificial serum have been given to feeble patients in twenty-four hours, and, according to the author, the results have declared themselves immediately. In addition the new solution can be administered without causing either pain or inflammation. It can be employed alike in acute and chronic infective maladies. Of the latter the most suitable for its exhibition are arthritis deformans and gout.

### The Abdominal Binder.

The time-honoured custom of binding the abdomen after labour comes in for severe criticism by H. M. Little in the *Journal of the Canadian Medical Association*. He does not of course hold the binder responsible for the evil results following an ill-managed puerperium, but he believes that it has a distinct influence on adverse conditions arising from labour. According to him it militates against a proper involution of the round ligaments which incline to draw the uterus forward. It prevents the uterus falling anteriorly on to the bladder and so tends to prohibit spontaneous micturition. Lastly, it keeps the uterus permanently held back so that the anterior cervical lip is held apart from the posterior. Consequently if lacerations are present they are unable to heal directly and scar tissue is formed in the angles of the wound. This permanent gaping of the cervical lips causes discomfort later and may be associated with a permanent backward displacement of the uterus. In cases in which no binder is used it is possible to obtain a clear idea of the progress of uterine involution, and the abdominal muscles get much freer play with consequent improvement in their condition of tone.

### Ultimate Results in Shoulder-dislocations.

Some attention has lately been devoted in the French and German medical press to the prognosis of cases of dislocation of the shoulder-joint after reduction. Authorities seem agreed that it is always best to give a guarded opinion even in cases where an examination with the $x$-rays has shown that the bony skeleton has suffered no perceptible damage. Out of every hundred cases not more than fifteen regain complete functional activity. The remainder present varying degrees of reduced muscular activity, limitation of movement, articular crackling and pain. Lenormant in the *Presse Médicale* states that the majority of old cases of dislocation of the shoulder undergo by the very fact of the accident a partial and definite diminution of their functional value. This diminution averages about 20 per cent. but may attain 50 per cent. in about one-tenth of cases. Most cases show a few months later a notable atrophy of the periarticular muscles. There is relative impotence with limitation of movement, especially in the motion of abduction. The arm cannot be raised to the horizontal position, and the power of abduction is reduced one half. Palpation reveals crackling in the joint. This atrophy and stiffness is caused frequently by bony and nervous lesions. Radiography may show that the dislocation is complicated by the tearing away of portions of the tuberosities of the humerus; after consolidation the detached fragments may form callus on the borders of the glenoid cavity and so limit abduction. The nervous lesions are due to compression and stretching which may lead to circumflex paralysis. In many cases, however, neither bony nor nervous lesions exist to explain the condition of impaired function. The pathology of such injuries remains obscure. All cases should therefore be $x$-rayed after immediate reduction, the limb should be immobilised for forty-eight hours only, after which it should be moved freely, and a not too hopeful prognosis given.

